# Validation of the China mortality prediction model in trauma based on the ICD-10-CM codes

**DOI:** 10.1097/MD.0000000000038537

**Published:** 2024-06-21

**Authors:** Zi-Xiao Zhang, Yan-Hua Wang, Zhong-Di Liu, Tian-Bing Wang, Wei Huang

**Affiliations:** a Trauma Medicine Center, Peking University People’s Hospital, Beijing, China; b Department of Traumatology and Orthopedics, Peking University People’s Hospital, Beijing, China.

**Keywords:** mortality risk prediction model, trauma, validation

## Abstract

The China mortality prediction model in trauma, based on the International Classification of Diseases, Tenth Revision, Clinical Modification lexicon (CMPMIT-ICD-10), is a novel model for predicting outcomes in patients who experienced trauma. This model has not yet been validated using data acquired from patients at other trauma centers in China. This retrospective study used data retrieved from the Peking University People’s Hospital discharge database and included all patients admitted for trauma between 2012 and 2022 for model validation. Model performance was categorized into discrimination and calibration. In total, 23,299 patients were included in this study, with an overall mortality rate of 1.2%. CMPMIT-ICD-10 showed good discrimination and calibration, with an area under the curve of 0.84 (95% confidence interval: 0.82–0.87) and a Brier score of 0.02. The performance of the CMPMIT-ICD-10 during validation was satisfactory, and the application of the model will be scaled up in future studies.

## 1. Introduction

Trauma mortality prediction models are essential for trauma patient care, trauma-related clinical research, and the assessment of the quality of trauma care. Currently, the mortality risk from trauma is assessed using 2 types of models. The first type includes Abbreviated Injury Scale (AIS)-based models such as the Injury Severity Score (ISS), Trauma and ISS, and Revised Injury Severity Classification II.^[1–3]^ The second type includes models based on codes from the International Classification of Diseases, Tenth Revision, Clinical Modification lexicon (ICD-10-CM) such as the Trauma Mortality Prediction model based on ICD-10-CM Codes, Injury Mortality Prediction, and ICD-10-based ISS.^[4,5]^ Although ISS has been widely used, coders require specialized training to use complex AIS codes, which is time- and resource-consuming.^[5,6]^ Furthermore, most Chinese databases do not have separate AIS codes, whereas ICD-10-CM codes are widely used. The trauma mortality prediction model based on ICD-10-CM codes is relatively complex and does not account for factors such as previous comorbidities or acute physiological reactions. Therefore, to overcome the limitations of existing trauma mortality prediction models, Wang et al developed the China mortality prediction model for trauma based on ICD-10-CM codes (CMPMIT-ICD-10), which was the first prediction model established specifically for Chinese patients who experience trauma.^[7]^ The predictor variables in the CMPMIT included sex, age, ICD-10-CM codes, comorbidities, post-traumatic physiological responses, and state of consciousness. CMPMIT has shown excellent performance in development datasets and internal validations; however, it has yet to be validated outside the developing environment.

## 2. Methods

### 2.1. Participants

Peking University People’s Hospital is responsible for the treatment and care of trauma patients in Beijing and the surrounding areas. The hospital collects data on post-trauma patients from its discharge database, including patient demographic characteristics, ICD-10-CM codes, state of consciousness, mechanism of injury, treatment, expenses, and outcomes at discharge.

We included all patients admitted to the hospital for traumatic events between January 2012 and May 2022 as the study population. Traumatic events were those listed in Chapter 20 of the ICD-10-CM, excluding hanging, asphyxia, drowning, poisoning, burns, and electrocution. Patients with missing baseline characteristics or outcomes were excluded. This was a retrospective, non-interventional study based on anonymous registry data.

### 2.2. Statistical methods

The mortality risk associated with CMPMIT was calculated as described in our previous study.



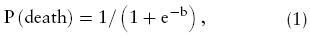



where P (death) is the probability of death and e is the constant 2.718282.

The value of b was calculated from the following equation:



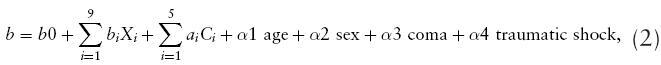



where Xi, i = 1...9 represents the 9 region-severity codes included in the model as the dichotomous variables, Ci, i = 1...5 represents the dichotomous variables for each comorbidity, “sex,” “coma,” and “traumatic shock” are dichotomous variables, with “male” as the reference group in sex, and “age” is a multicategorical variable. Mortality at discharge was considered as the outcome.

R Studio 4.0.2 and SPSS v24.0.0.1 (IBM Corporation, Armonk) were used for statistical analysis. The variables included in the study were subjected to a univariate analysis. We used the chi-square test or Fisher exact test for categorical univariate analyses. We used the Mann–Whitney *U* test or Student *t* test for continuous data.

In this study, discrimination and calibration were used to assess the model performance. Discrimination assesses a model’s ability to distinguish between deceased and surviving patients. The area under the receiver operating characteristic curve (AUC) can be used as a measure of discrimination, with an AUC closer to 1 indicating better discrimination. Generally, discrimination with an AUC > 0.90 is considered excellent, AUC > 0.80 is considered good, and AUC < 0.70 is considered poor. Discrimination is a key concern when assessing the model performance.^[8]^

Calibration reflects the accuracy of the model’s absolute value of risk prediction; that is, the degree to which the model’s predicted probability agrees with the observed probability of an event occurring. The Brier score was used as a measure of calibration and was calculated as follows:



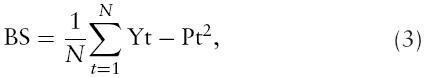



where Yt is the actual observed value of the variable indicating the outcome (0 or 1) and Pt is the prediction probability given by the prediction model. A Brier score closer to 0 indicated better model calibration and a Brier score > 0.25 suggests that the model had no predictive power.^[9]^

## 3. Results

During the study period, 23,299 trauma patients were included, with 286 deaths. The mean age of the patients was 58 years, 10,887 (46.7%) were men, and the overall mortality rate was 1.2%. The baseline patient characteristics are shown in Table 1.

**Table 1:** Baseline characteristics of patients included in the study (2012–2022).

Characteristics	Total population	Survival	Mortality	*P* value
	(N = 23,299, 100%)	(N = 23,013, 98.8%)	(N = 286, 1.2%)	
Age, mean (SD)	58.2 (19.1)	58.1 (20.3)	70.5 (18.6)	<.001
Male sex	10,887 (46.7%)	10,729 (46.6%)	158 (55.2%)	<.001
Coma	40 (0.2%)	28 (0.1%)	12 (4.2%)	<.001
Traumatic shock	247 (1.1%)	187 (0.8%)	60 (21.0%)	<.001
A2	283 (1.2%)	268 (1.2%)	15 (5.2%)	<.001
A3	475 (2.0%)	447 (1.9%)	28 (9.8%)	<.001
A4	499 (2.1%)	468 (2.0%)	31 (10.8%)	<.001
A5	13 (0.1%)	10 (0.0%)	3 (1.0%)	<.001
D3	317 (1.4%)	296 (1.3%)	21 (7.3%)	<.001
E2	278 (1.2%)	261 (1.1%)	17 (5.9%)	<.001
E3	120 (0.5%)	116 (0.5%)	4 (1.4)	.036
F2	1805 (7.7%)	1781 (7.7%)	24 (8.4%)	.682
G3	4203 (18.0%)	4124 (17.9%)	79 (27.6%)	<.001
Coronary heart disease	2209 (9.5%)	2116 (9.2%)	93 (32.5%)	<.001
Heart failure	893 (3.8%)	804 (3.5%)	89 (31.1)	<.001
Renal failure	623 (2.7%)	537 (2.3%)	86 (30.1%)	<.001
Cerebrovascular disease	2863 (12.3%)	2765 (12.0%)	98 (34.3%)	<.001
Peptic ulcer	378 (1.6%)	352 (1.5%)	26 (9.1%)	<.001

A2–G3 are the new region-severity code.

**Figure 1.**
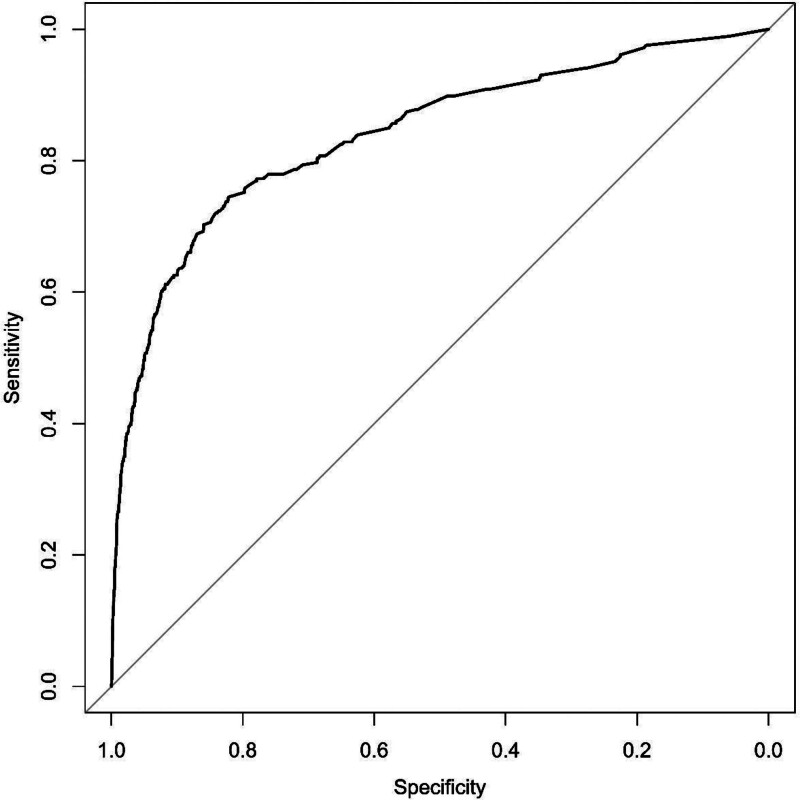
Receiver operating characteristic (ROC) curve for validation of China mortality prediction model in trauma based on International Classification of Diseases, Tenth Revision, Clinical Modification codes (CMPMIT-ICD-10).

CMPMIT showed good discrimination, with an AUC of 0.84 (95% confidence interval: 0.82–0.87), and the receiver operating characteristic curve is shown in Figure 1. Calibration of the model was excellent with a Brier score of 0.02, indicating that the observed mortality rate was close to the predicted value.

## 4. Discussion

Currently, most trauma scoring systems are based on databases from developed countries; however, most trauma cases occur in developing countries.^[10–12]^ Medical resources are relatively scarce in developing countries. Thus, early and accurate prediction of mortality can provide information for patient care stratification to ensure the appropriate allocation of medical resources.^[13]^ Owing to differences in medical standards and resources, a distinct trauma scoring system should be developed for developing countries to ensure effective triage.^[14]^

As the cost of medical care for trauma treatment increases, assessing the quality of care across trauma treatment facilities becomes increasingly important. This requires the adjustment of post-trauma mortality risk to be as accurate as possible.^[15]^ Comparing crude mortality rates across hospitals is meaningless without first adjusting for differences in patient composition, as the observed differences in mortality rates may simply be the result of differences in the trauma patient population across hospitals.^[16]^ As different hospitals have different patient compositions, mortality risk adjustment levels the playing field and makes the performance of different hospitals comparable. Therefore, there is a need for validated mortality prediction models that can be used to adjust for differences in patient composition and disease severity across hospitals.

Developing trauma mortality prediction models is challenging because thousands of injury types and combinations exist. However, predicting many trauma combinations is challenging. Currently, the most widely applied ISS model addresses this problem to some extent by calculating the AIS score squared for the 3 most severely injured body regions.^[17]^ However, inconveniently, ISS- and ISS-based trauma mortality prediction models can only be used when injuries are described using AIS codes. Injury descriptions require trained injury coders to review medical records and parse each injury into an AIS code. However, this process is time-consuming and expensive.

In China, AIS injury coding is performed only in some trauma centers. Unlike AIS codes, ICD-10 codes are commonly assigned to all injuries sustained by inpatients with trauma. Thus, a trauma mortality prediction model based on ICD-10 codes allows the prediction of mortality without recoding.

For these reasons, in our previous study, we developed the CMPMIT-ICD-10, the first trauma mortality prediction model based on Chinese trauma databases and ICD-10 codes.^[4]^ Compared to other trauma mortality prediction models based on ICD-10 codes, which only focus on injuries, CMPMIT-ICD-10 focuses more on the overall condition of patients with trauma. The influence of comorbidities and post-traumatic physiological responses on mortality was further considered in this new model. In addition, the model is simple and easy to use. Under the current trauma care system in China, it is suitable for early risk stratification of trauma patients and quality assessment of trauma centers. However, since its establishment, the CMPMIT-ICD-10 has not yet been validated with other databases in terms of portability and generalizability. In this study, we validated the CMPMIT-ICD-10 using the discharge database of Peking University People’s Hospital. Overall, the validation results were satisfactory. The AUC generated during model validation was 0.84, which indicated that the performance was not as good as that during validation using the development dataset but also that the model had relatively good discrimination. The Brier score for the model validation was 0.02, which was similar to that for validation using the development dataset, indicating that the model had good calibration. These results indicate that this novel model is portable and generalizable.

The following reasons may explain why the model did not perform as well in this validation, as in the validation using the developed dataset. In this model, many ICD-10 codes share a common coefficient after reclassification, which may not be an accurate measurement of trauma severity. Moreover, post-trauma biochemical indicators such as hemoglobin and base deficit were not included in the model, which may have led to a less accurate prediction of mortality risk.^[8,18]^

The biggest limitation of this study is that the validation database was a single-center database and had low mortality rates despite its large sample size. Results from the validation using this database may not accurately reflect the model’s ability to predict mortality risk in a severely traumatized population. In addition, machine learning techniques have emerged as powerful tools for predictive modeling in recent years, including in the trauma domain.^[6,19–24]^ However, it is still unclear whether machine learning models or traditional logistic regression models are more effective and accurate in predicting the mortality of trauma patients. Owing to the limitations of our database, we were unable to compare this model with existing machine learning models.

This model must be validated in numerous settings in future studies. In addition, the model must be continuously refined using new data and methods.

## 5. Conclusions

The CMPMIT-ICD-10 showed good discrimination and calibration in the validation; therefore, it will be improved and applied on a larger scale in the Chinese population.

## Author contributions

**Conceptualization:** Zi-Xiao Zhang, Wei Huang.

**Data curation:** Yan-Hua Wang.

**Formal analysis:** Zhong-Di Liu.

**Methodology:** Yan-Hua Wang.

**Validation:** Tian-Bing Wang, Wei Huang.

**Writing – original draft:** Zi-Xiao Zhang.

**Writing – review & editing:** Zi-Xiao Zhang, Wei Huang.

## References

[1] Baker SP O’Neill B Haddon W Jr Long WB. The injury severity score: a method for describing patients with multiple injuries and evaluating emergency care. J Trauma. 1974;14:187–96. 4814394

[2] Liu XY Qin YM Tian SF. Performance of trauma scoring systems in predicting mortality in geriatric trauma patients: comparison of the ISS, TRISS, and GTOS based on a systemic review and meta-analysis [published online ahead of print February 16, 2004]. Nat Med. doi: 10.1007/s00068-024-02467-1. 38363328

[3] Halvachizadeh S Störmann PJ Özkurtul O. Discrimination and calibration of a prediction model for mortality is decreased in secondary transferred patients: a validation in the TraumaRegister DGU. BMJ Open. 2022;12:e056381. 10.1136/bmjopen-2021-056381 PMC9014053 35418430

[4] Osler TM Glance LG Cook A Buzas JS Hosmer DW. A trauma mortality prediction model based on the ICD-10-CM lexicon: TMPM-ICD10. J Trauma Acute Care Surg. 2019;86:891–5. 30633101 10.1097/TA.0000000000002194

[5] Wang M Qiu W Zeng Y Fan W Lian X Shen Y. IMP-ICDX: an injury mortality prediction based on ICD-10-CM codes. World J Emerg Surg. 2019;14:46. 31632453 10.1186/s13017-019-0265-y PMC6787998

[6] Choi J Vendrow EB Moor M Spain DA. Development and validation of a model to quantify injury severity in real time. JAMA Netw Open. 2023;6:e2336196. 37812422 10.1001/jamanetworkopen.2023.36196 PMC10562944

[7] Wang YH Wang TB Zhang ZX. Development and internal validation of China mortality prediction model in trauma based on ICD-10-CM lexicon: CMPMIT-ICD10. Chin Med J (Engl). 2021;134:532–8. 33560666 10.1097/CM9.0000000000001371 PMC7929565

[8] Lefering R Huber-Wagner S Nienaber U Maegele M Bouillon B. Update of the trauma risk adjustment model of the TraumaRegister DGU™: the revised injury severity classification, version II. Crit Care. 2014;18:476. 25394596 10.1186/s13054-014-0476-2 PMC4177428

[9] Yang W Jiang J Schnellinger EM Kimmel SE Guo W. Modified Brier score for evaluating prediction accuracy for binary outcomes. Stat Methods Med Res. 2022;31:2287–96. 36031854 10.1177/09622802221122391 PMC9691523

[10] Leilei D Pengpeng Y Haagsma JA. The burden of injury in China, 1990-2017: findings from the global burden of disease study 2017. Lancet Public Health. 2019;4:e449–61. 31493842 10.1016/S2468-2667(19)30125-2 PMC6739690

[11] Hu X Qi M Yuan P. Disease burden evaluation of injury and poisoning in China from 2009 to 2019. Iran J Public Health. 2023;52:986–94. 37484713 10.18502/ijph.v52i5.12717 PMC10362209

[12] Hung KCK Lai CY Yeung JHH. RISC II is superior to TRISS in predicting 30-day mortality in blunt major trauma patients in Hong Kong. Eur J Trauma Emerg Surg. 2022;48:1093–100. 33900416 10.1007/s00068-021-01667-3

[13] Akay S Ozturk AM Akay H. Comparison of modified Kampala trauma score with trauma mortality prediction model and trauma-injury severity score: a national trauma data bank study. Am J Emerg Med. 2017;35:1056–9. 28222915 10.1016/j.ajem.2017.02.035

[14] Lam SW Lingsma HF van Beeck EF Leenen LP. Validation of a base deficit-based trauma prediction model and comparison with TRISS and ASCOT. Eur J Trauma Emerg Surg. 2016;42:627–33. 26555726 10.1007/s00068-015-0592-y

[15] Cook A Osler T Glance L. Comparison of two prognostic models in trauma outcome. Br J Surg. 2018;105:513–9. 29465764 10.1002/bjs.10764

[16] Haider AH Villegas CV Saleem T. Should the IDC-9 trauma mortality prediction model become the new paradigm for benchmarking trauma outcomes? J Trauma Acute Care Surg. 2012;72:1695–701. 22695443 10.1097/TA.0b013e318256a010

[17] Copes WS Champion HR Sacco WJ Lawnick MM Keast SL Bain LW. The injury severity score revisited. J Trauma. 1988;28:69–77. 3123707 10.1097/00005373-198801000-00010

[18] Brockamp T Maegele M Gaarder C. Comparison of the predictive performance of the BIG, TRISS, and PS09 score in an adult trauma population derived from multiple international trauma registries. Crit Care. 2013;17:R134. 23844754 10.1186/cc12813 PMC4057174

[19] Dijkstra H van de Kuit A de Groot T. Systematic review of machine-learning models in orthopaedic trauma. Bone Jt Open. 2024;5:9–19. 38226447 10.1302/2633-1462.51.BJO-2023-0095.R1 PMC10790183

[20] Cardosi JD Shen H Groner JI Armstrong M Xiang H. Machine learning for outcome predictions of patients with trauma during emergency department care. BMJ Health Care Inform. 2021;28:e100407. 10.1136/bmjhci-2021-100407 PMC8504344 34625448

[21] Khalili H Rismani M Nematollahi MA. Prognosis prediction in traumatic brain injury patients using machine learning algorithms. Sci Rep. 2023;13:960. 36653412 10.1038/s41598-023-28188-w PMC9849475

[22] Gorczyca MT Toscano NC Cheng JD. The trauma severity model: An ensemble machine learning approach to risk prediction. Comput Biol Med. 2019;108:9–19. 30965177 10.1016/j.compbiomed.2019.02.025

[23] Yang S Cao L Zhou Y Hu C. A Retrospective cohort study: predicting 90-day mortality for ICU trauma patients with a machine learning algorithm using XGBoost using MIMIC-III database. J Multidiscip Healthc. 2023;16:2625–40. 37701177 10.2147/JMDH.S416943 PMC10493110

[24] Cao Y Forssten MP Sarani B Montgomery S Mohseni S. Development and validation of an XGBoost-algorithm-powered survival model for predicting in-hospital mortality based on 545,388 isolated severe traumatic brain injury patients from the TQIP database. J Pers Med. 2023;13:1401. 37763168 10.3390/jpm13091401 PMC10533165

